# Metagenetic Analysis of the Pregnant Microbiome in Horses

**DOI:** 10.3390/ani13121999

**Published:** 2023-06-15

**Authors:** Kalie F. Beckers, Viviane C. L. Gomes, Kassandra R. Crissman, Chin-Chi Liu, Christopher J. Schulz, Gary W. Childers, Jenny L. Sones

**Affiliations:** 1 Veterinary Clinical Sciences, School of Veterinary Medicine, Louisiana State University, Baton Rouge, LA 70803, USA; kbeck11@lsu.edu (K.F.B.);; 2 Department of Biological Sciences, Southeastern Louisiana University, Hammond, LA 70402, USA

**Keywords:** equine, placenta, reproductive tract, microbiome

## Abstract

**Simple Summary:**

This study aims to identify the microbial communities in different body sites of the pregnant mare in early gestation to describe core microbiomes for each. Samples were collected from the oral cavity, vagina, anus, and the allantoic portion of the allantochorion (“placenta”) from five pregnant mares between 96 and 120 days of gestation. The V4 region of the 16S rRNA gene was amplified for Illumina MiSeq sequencing to examine core bacterial communities present in the different body sites. The placenta was significantly different from feces, oral cavity, and the vagina. Alpha diversity measuring the Shannon diversity matrix was significant, with the body sites being a compounding variable, meaning there was a difference in richness and evenness in the different microbial communities. Feces had the greatest alpha diversity, while the oral cavity and placenta similarly had the least. The microbial communities of the equine placenta show similarities at the genus level to the oral cavity, both harboring *Gemella* and *Porphyromonas*. In conclusion, metagenetics did reveal distinct community differences between the oral cavity, feces, vagina, and placenta of the pregnant horse.

**Abstract:**

Placentitis is the leading cause of infectious abortion in the horse. Additionally, it can result in weak and/or growth restricted offspring. While the etiology of ascending placentitis is well described in mares, less is known regarding the pathogenesis of other types, such as nocardioform placentitis. This study aims to identify the microbial communities in different body sites of the pregnant mare in early gestation to establish a core microbiome that may be perturbed in pathologic pregnancies such as placentitis. We hypothesize that the equine placenta harbors a distinct resident microbiome in early pregnancy when characterized by metagenetics and that there will be a disparity in bacterial communities from the oral, vaginal, and fecal microbiome. Samples were collected from the oral cavity, vagina, anus, and the allantoic portion of the allantochorion (“placenta”) from five pregnant mares between 96 and 120 days of gestation. The V4 region of the 16S rRNA gene was amplified for Illumina MiSeq sequencing to examine core bacterial communities present in the different body sites. Microbial community composition of the pregnant ponies by body site was significantly different (Bray–Curtis dissimilarity). The placenta was significantly different from the feces, oral cavity, and vagina. Alpha diversity measuring the Shannon diversity matrix was significant, with the body sites being a compounding variable, meaning there was a difference in richness and evenness in the different microbial communities. Feces had the greatest alpha diversity, while the oral cavity and placenta similarly had the least. In conclusion, metagenetics did reveal distinct community differences in the oral, fecal, vaginal, and placenta cavities of the horse. The equine placenta does show similarities in its microbial communities to the oral cavity. Further research needs to be completed to investigate how bacteria may be translocated to the placenta from these other body sites and how they contribute to the development of placentitis.

## 1. Introduction

The placenta is a transient yet vital organ of pregnancy. It was long thought to be sterile, and this dogma has been challenged in the past decade. The placenta is necessary to maintain a healthy pregnancy. First, proper growth and function of the placenta are essential for development of the fetus [1]. The placenta ensures the exchange of nutrients and waste products between the maternal and fetal circulatory systems [2,3]. The placenta is also a hormone-secreting tissue for pregnancy maintenance and immunotolerance of the semi-allogeneic fetus. Therefore, dampened maternal immune responses align with the idea that the placenta is sterile. Recently, a controversial theory of the placental microbiome in humans has been reported [4,5]. This represents a novel function of the local maternal milieu and pregnancy success. The initial discovery of the human placental microbiome was made using next-generation metagenomic sequencing techniques [6]. The microbiome is the combined genetic material of the microorganisms in a specific environment or body site, while metagenetics is the study of genes expressed by microorganisms recovered from a specific site. The host and microbiome relationship are considered to be in mutualistic symbiosis [7]. Studies have shown that the human body provides the sustenance for the microbes and, in return, the microbes execute essential functions for the host [8,9]. When the composition of the microbial environment is perturbed, disease can occur [10,11,12]. This is referred to as microbial dysbiosis and can be defined as a combination of a reduction in microbial diversity and the loss of beneficial bacteria [13]. An example of dysbiosis in humans is a different placental microbiome being detected in normal pregnancies compared with pregnancies complicated by preterm birth [14]. A similar scenario may occur in horses, but further research needs to be completed to identify a healthy maternal reproductive microbiome during pregnancy, which is then compared with the diseased state. 

Similar to other eutherian mammals, the placenta of the horse comprises several fetal membranes. The equine placenta is made up of two distinct membranes: (1) the amnion, the opaque membrane that immediately surrounds the foal, and (2) the chorioallantois, which interdigitates with the endometrium on the chorionic side [15]. The equine placenta is classified as diffuse, meaning the entire surface of the uterus is covered by the chorioallantois. A small area adjacent to the cervix, called the “cervical star”, has no chorionic attachment because it is lacking an endometrium [16]. The placental microbiome of the horse has not yet been well described. The rationale to investigate the equine placental microbiome comes from non-pregnant reproductive tract studies described in the horse [17,18,19]. The non-pregnant mare reproductive tract may influence the microbiome of the developing placenta. A distinct uterine microbiome has been reported in diestrual mares (day 7 after ovulation) carrying an embryo compared with those that are open using metagenetics [17]. Proteobacteria and Bacteroidetes are associated with culture-positive samples at ovulation. *Sphingobium* (Proteobacteria) and Sphingobacteriales (Bacteroidetes) are associated with mares carrying embryos at day 7 post ovulation, and Rhodocyclaceae and Enterobacteriaceae (Proteobacteria) are associated with mares not carrying embryos [17]. Amniotic fluid taken at delivery from healthy equine pregnancies has also yielded bacterial growth [20]. Additionally, foal meconium on postnatal day 1 is dominated by the Firmicutes phyla consisting primarily of the genera *Enterococcus, Bacillus*, and *Lactococcus* [21,22].

It has previously been shown that the equine placenta collected after vaginal delivery from healthy mares harbors a unique microbiome [23,24]. It is unknown how this may contribute to pregnancy outcomes in horses. This study aims to identify the core microbial communities in different body sites of the pregnant mare in the first third of gestation. We hypothesize that the equine placenta harbors a distinct resident microbiome in early pregnancy when characterized by metagenetics, and that there will be a disparity in bacterial communities from the oral, vaginal, and fecal microbiome. Furthermore, we believe metagenetics will reveal distinct communities and indicator taxa that characterize a healthy equine placenta. Understanding the core microbiome that may be perturbed in pathologic pregnancies will improve our understanding of this disease in mares. 

## 2. Materials and Methods

### 2.1. Animal Experiments

Five pregnant pony mares (age 4–8 years old) were used between 96 and 120 days of gestation upon necropsy, see Table 1. The pony mares underwent a health evaluation and were monitored by ultrasound by a board-certified theriogenologist throughout pregnancy. All pony mares were co-housed and naturally serviced by the same pony stallion. After euthanasia, swabs were sterilely collected from the oral cavity, vagina, rectum, and the allantoic portion of the allantochorion. The uterus was removed in situ to maintain sterility during sample collection. Briefly, the whole reproductive tract was removed by incising ovarian ligaments and the vagina distal to the cervix, keeping the cervix closed, and this was placed on a separate tray and table. The uterus was incised adjacent to the conceptus with a sterile scalpel blade and the entire conceptus was detached from the endometrium. The swab was inserted into the allantoic cavity and rotated on the surface to collect the allantoic sample. The allantoic swab will be referred to as the placental sample in the following sections. Samples were placed in a sterile tube and stored at −80 °C until further analysis. The blank samples were swabs taken from the environment by briefly exposing them to the environment in which the samples were collected. The blank swabs were immediately frozen and processed the same way as the body site samples. Samples were collected in accordance with Louisiana State University School of Veterinary Medicine IACUC regulation for deceased sample collection, approval number 17-020.

### 2.2. DNA Sequencing

Microbial DNA was extracted from different body site samples using the Qiagen DNeasy PowerSoil extraction kits (Qiagen, Germantown, MD, USA) according to manufacturer’s protocol. Samples that did not have enough biomass were not able to be sequenced. The V4 variable region of the 16S rRNA gene was amplified with PCR primers 515f/806r [25] in a 30 cycle PCR using the DreamTaq Hot Start PCR Master Mix Kit (Thermoscientific, Waltham, MA, USA). PCR was performed in 20 μL vol and included 2 μL (7.5 μM concn) of forward and reverse primers, 12.5 μL of Hot Start Taq 2X Master Mix (New England BioLabs Inc., Ipswich, MA., USA), 3.5 μL of deionized water, and 2 μL of sample DNA. Thermal cycle conditions were 95 °C for 3 min for the initial denaturing step, followed by 30 cycles of 95 °C for 30 s, 50 °C for 1 min, and 72 °C for 1 min. PCR products were checked on a 2% agarose gel for correct product size formation (approx. 350 bp). The Michigan State University Genomics Core performed library preparation prior to Illumina MiSeq sequencing, following the manufacturer’s guidelines [25]. Reagent controls using certified DNA free water were run through library preparation and PCR and did not generate libraries. For quality control, samples submitted for sequencing included a random blank sample of technical replicates. 

### 2.3. Bioinformatics and Statistics

Initial quality screening, demultiplexing, amplicon sequence variant (ASV) inference, and chimera removal were performed using the DADA2 package [26]. The decontam package was used to discern between the true bacterial sequences and potential contaminant DNA [27]. Given the nature of this study, low microbial biomass characterization requires contaminant removal to ensure that DNA from biological samples can be effectively distinguished from environmental blank contaminates and exogenous DNA. ASVs were classified using the Silva Release 132 16S rRNA database [28,29]. Microbial community analysis (alpha diversity, ASV richness, beta diversity, and beta dispersion) was performed using the vegan R package [30]. Permutational multivariate analysis of variance (PERMANOVA) [31] was performed using the vegan package Adonis function. To determine differentially abundant ASVs, the ASV table was first trimmed to only include ASVs with a median abundance greater than two across all samples. We applied a probabilistic framework, SourceTracker [32], to assess whether the microbial communities from the placenta appeared to source from other body site microbial community compositions. All statistical analyses were performed with JMP Pro 16.2.0 (SAS Institute Inc., Cary, NC, USA). Graphs were generated using Prism 9 for Windows, Version 9.5.0 (GraphPad software, LLC, San Diego, CA, USA). Relative abundance and alpha diversity were analyzed via mixed ANOVA and post-hoc Tukey tests with site as the fixed effect and each horse as the random effect. Logarithmic transformation was performed for data that did not meet the normality criteria. Normality of residuals from the models were accessed and confirmed via Shapiro–Wilk tests and quantile–quantile (Q–Q) plots. Data are presented as mean ± SD. Significance was set at *p* < 0.05. All raw sequence reads, and corresponding metadata, can be found on the SRA website accession number SUB12947298. 

## 3. Results

### 3.1. Alpha Diversity Using Shannon Diversity Matrix Was Significantly Different between Body Sites

**Figure 1:**
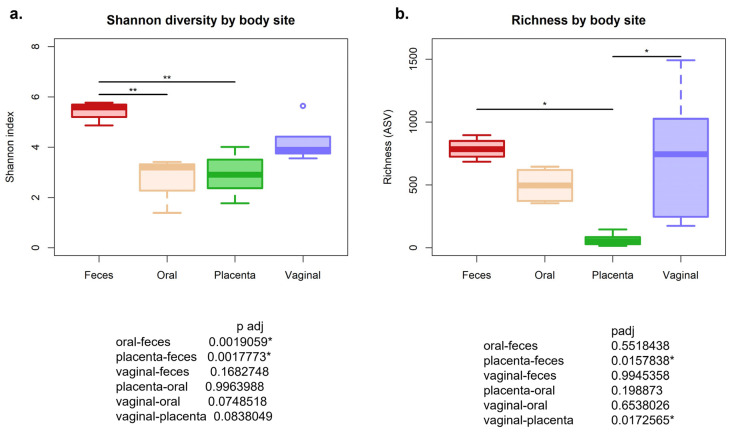
(**a**) Alpha diversity measuring the Shannon diversity was significant, with the different body sites being a compounding variable (*p* = 0.0008). (**b**). ASV richness was also significantly different by body site. * *p* < 0.05, ** *p* < 0.01.

Of all the 20 samples processed and sequenced, 3 placenta samples demonstrated low sequence reads. There was an overall average of 220,030 reads from all body sites after quality filtering. After the use of the decontam package in R, only 47 contaminated reads were found using the frequency method, while no contaminated reads were found using the prevalence method. When assessing the microbial communities using alpha diversity, the Shannon diversity matrix was significant, with the body sites being a compounding variable (*p* = 0.0008). Therefore, there was a difference in richness and evenness in the different microbial communities by site. When using Tukey’s multiple comparisons, it was found that the placenta diversity index (2.91 ± 0.89) was not significantly different from the oral cavity (2.79 ± 0.95) when assessing alpha diversity alone (*p* = 0.8297). Feces was found to be the most diverse (5.45 ± 0.40), followed by the vagina (4.25 ± 0.84), while the oral cavity and placenta were the least (oral–feces *p* = 0.0051, placenta–feces *p* = 0.0206; Figure 1a). When assessing only ASV richness, the placental samples were significantly less rich compared with vaginal (*p* = 0.016) and fecal samples (*p* = 0.017) (Figure 1b).

### 3.2. Bacterial Community Compositions Were Different among Body Sites When Assessing Beta Diversity

**Figure 2:**
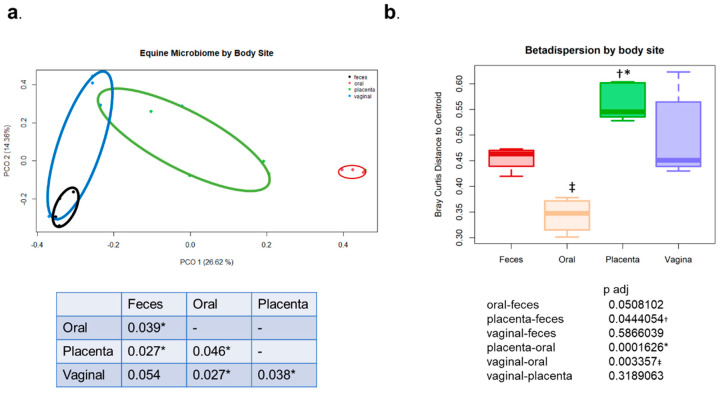
(**a**) Beta diversity of equine microbiome by body site using PERMANOVA with Bray–Curtis dissimilarity of 16S amplicon sequence variant’s relative abundance. *p* = 0.001. (**b**). Beta dispersion of equine microbiome by different body sites. Using Tukey’s multiple comparison, * denotes significantly different from the oral cavity, † denotes significantly different from the feces, and ‡ denotes significantly different from the vagina.

Microbial community composition of the pregnant ponies by body site was significantly different when assessing beta diversity (*p* = 0.001, PERMANOVA with Bray–Curtis dissimilarity of 16S amplicon sequence variant’s relative abundance). The placenta was significantly different from feces (*p* = 0.027), the oral cavity (*p* = 0.046), and the vagina (*p* = 0.038) using Bray–Curtis dissimilarity (Figure 2a). Bray–Curtis dissimilarity is a commonly used assessment of beta diversity within the microbiology field. It quantifies the overall taxonomic composition between groups, in this case body sites. Bray–Curtis dissimilarity examines the abundance of microbes shared between samples. The dissimilarity ranges from 0 to 1. If both samples share exactly the same microbes, their ‘dissimilarity’ is equal to zero. If no microbes are shared, their ‘dissimilarity’ is equal to one [33]. In the case of this study, the oral and placenta samples grouped separately, while the vaginal and feces samples had some overlap. This means the vaginal and feces samples shared more microbes than the oral and placental samples. When evaluating beta dispersion, the placental samples were significantly different from the feces and oral samples (Figure 2b). The vaginal samples also had a significant increase in beta dispersion compared with the oral cavity. Beta dispersion demonstrates that the placenta samples are more dissimilar to other placenta samples when compared with samples from other body sites when assessing individual variation. 

### 3.3. Difference in Relative Abundance Found at Phyla and Genus Level

**Figure 3:**
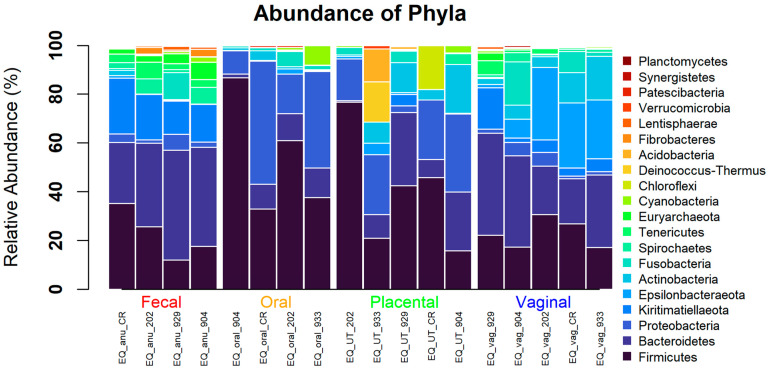
Relative abundance at the phyla level of equine microbiome at different body sites.

**Figure 4:**
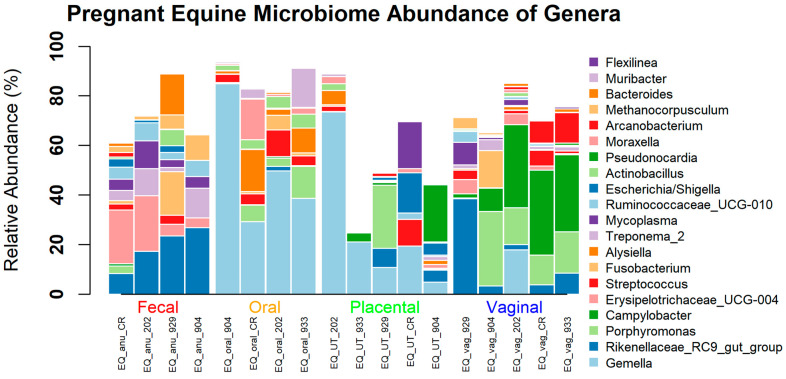
Relative abundance at the genus level of equine microbiome at different body sites.

**Figure 5:**
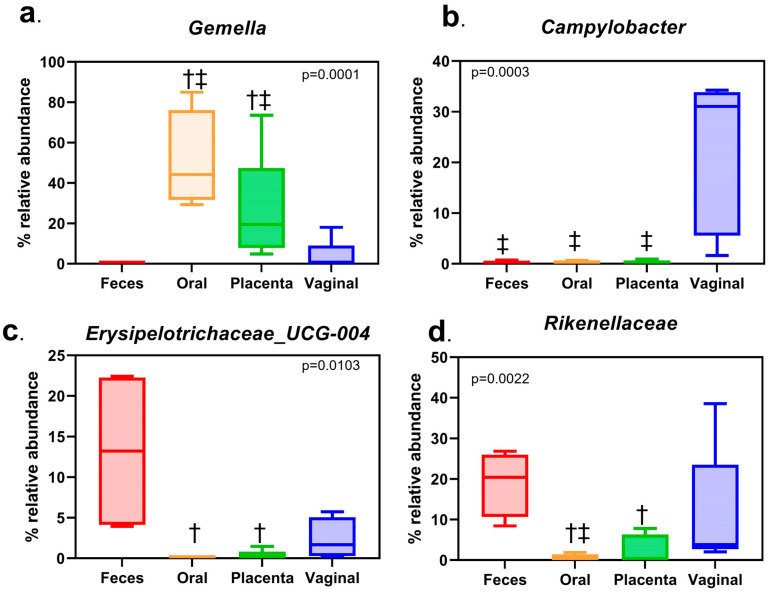
Top Genera that are different by body site. (**a**). Relative abundance differences by body site of *Gemella.* (**b**). Relative abundance differences by body site of *Campylobacter*. (**c**). Relative abundance differences by body site of *Erysipelotrichaceae_UCG*. (**d**). Relative abundance differences by body site of *Rikenellaceae*. Using Tukey’s multiple comparison, † denotes significantly different from the feces, and ‡ denotes significantly different from the vagina.

When evaluating the different body sites at the phyla level, all were dominated by Firmicutes, Bacteroidetes, Proteobacteria, and Actinobacteria (Figure 3). When analyzing at the genera level, the placenta was dominated by *Gemella*, *Rikenellaceae_RC9*, *Porphyromonas*, and *Streptococcus*. Similarly, the oral cavity was dominated by *Gemella*, *Porphyromonas*, *Streptococcus*, and *Alysiella*. The vagina consisted of *Rikenellceace_RC9*, *Porphyromonas*, *Campylobacter*, and *Streptococcus*. Finally, the fecal samples included *Rikenellaceae_RC9*, *Erysipelotrichaceae_UCG-004*, *Treponema_2*, and *Mycoplasma* (Figure 4). Only the top 20 of the most abundant genera were graphed. The remaining white space is made up of lesser taxa or unknown ASVs. The individual horse variation was not significantly different within the top genera analyzed. Using Tukey’s multiple comparison of the most abundant taxa, *Gemella* (*p* < 0.0001), *Campylobacteria* (*p* = 0.0003), *Rikenellaceae* (*p* = 0.0022)*,* and *Erysipelotrichaceae_UCG_004* (*p* = 0.0103) were different by body site (Figure 5)*,* while *Porphyromoas*, *Streptococcus*, *Alysiella*, *Fusobacterium*, and *Bacteroides* were not (*p* > 0.05). More specifically, *Gemella* was significantly increased in the oral and placental samples compared with the feces and vaginal samples. *Campylobacter* was decreased in the feces, oral, and placental samples compared with the vaginal samples. *Erysipelotrichaceae_UCG-044* was significantly decreased in the oral and placental samples when compared with feces, but not different in the vaginal samples. Finally, *Rikenellaceae* was lower in the oral samples compared with the feces and vaginal samples, while it was only significantly decreased in the placental samples when compared with feces. 

### 3.4. The Estimated Bacterial Source of the Placental Microbiome Is Mainly of Unknown Origin 

**Figure 6:**
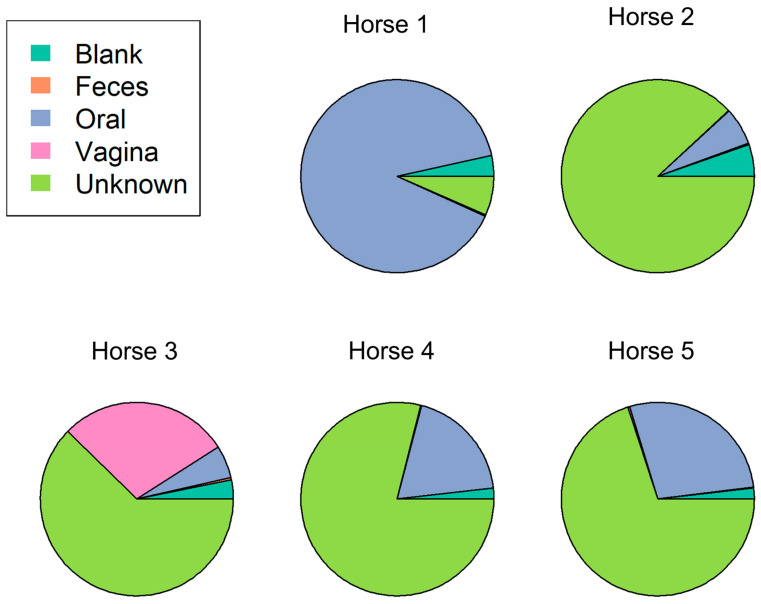
Pie chart of the estimated placental samples bacterial sources identified by SourceTracker package in R.

A typical microbial community comprises several source environments that could include contaminants from other bacterial communities or even contamination from sampling or the DNA extraction process. It is important in next generation sequencing studies to differentiate the environmental or iatrogenic contamination from naturally occurring source contamination. SourceTracker package in R was used to identify the potential source of the microbes found within the placental samples. This did vary by individual horse. For example, horse 1 placental samples were, for the majority, sourced from the oral cavity. Horses 2, 3, 4, and 5 were on the whole sourced from an unknown bacterial origin, followed by the oral cavity for horses 2, 4, and 5. Conversely, horse 3 secondary source was found to be from the vagina (Figure 6). Since the blanks taken in this study were included in this analysis, it demonstrates that the potential of contamination in these samples is low. The percentage of contribution of the blanks in the placental samples ranges from 1% to, at most, 5%, whereas the contribution from the oral cavity ranges from 5% to 89%. A remarkable finding was the percentage of bacterial sources categorized as “unknown” identified within the placental samples. The range of unknown source origins ranged from 6% to 88%, with the majority of the horses ranging from 62% to 78%. This highlights the importance of research in this field to identify these unknown microbes and how they are contributing to the development of the placental microbiome. 

### 3.5. Microbial Relatives of the Known Nocardioform Pathogens Were Found within These Healthy Horse Placentas

**Figure 7:**
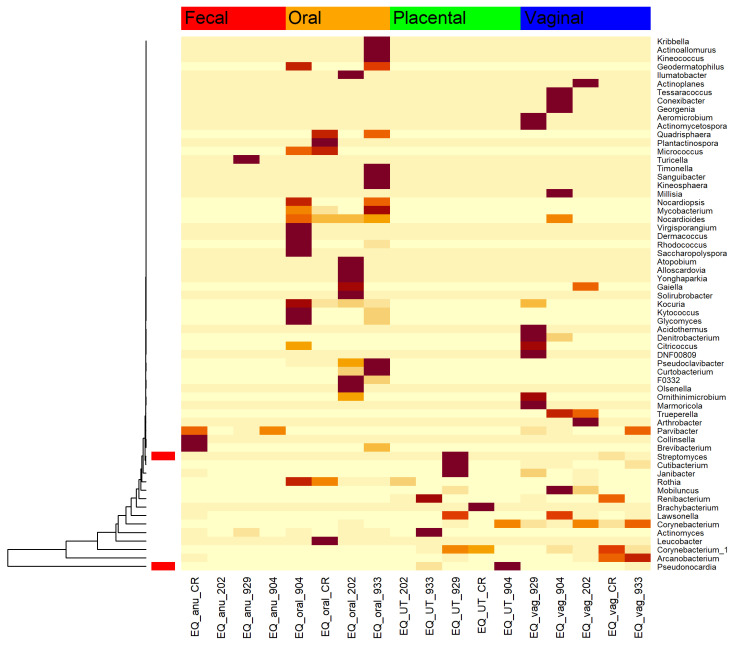
Heatmap of Actinobacteria phyla by body site. The red rectangles recognize the genera that are related to known nocardioform pathogens in the horse. The darker the coloration, the more abundant the taxa.

Known nocardioform pathogens are all members of the phyla Actinobacteria. These include *Amycolatopsis*, *Crossiella*, and *Streptomyces* [34]. A heatmap was used to visualize the members of Actinobacteria within these healthy pony pregnancies to investigate their abundances. Two members relating to known pathogens were identified: *Streptomyces* and *Pseudonocardia*. In this study, *Streptomyces* spp was isolated in one placental and one vaginal sample (Figure 7). *Pseudonocardia* spp, a close relative of *Amycolatopsis* and *Crossiella*, was isolated in two placental samples (Figure 7).

## 4. Discussion

The overarching goal of this study was to characterize the maternal microbiome of the pregnant horse by analyzing the placenta and extraplacental body sites, fecal, oral, and vaginal cavities, and to determine their differences. We hypothesize that the equine placenta harbors a distinct resident microbiome in early pregnancy when characterized by metagenetics and that there will be a disparity in bacterial communities from the oral, vaginal, and fecal microbiome. Furthermore, we believe metagenetics will reveal distinct communities and indicator taxa that characterize healthy placenta and extra-placental body sites in the mare. Main findings revealed that feces had the most diversity, while the oral and placental microbiome had the least. Oral and placental sites grouped differently when analyzed with beta diversity using Bray–Curtis dissimilarity, while vaginal and fecal microbiomes did not. At the genus level, some similarities were shown between body sites, even though the placenta did harbor its own unique microbiome in relation to diversity, relative abundance, and bacterial richness. The placenta was dominated by *Gemella*, *Rikenellaceae_RC9*, *Porphyromonas*, and *Streptococcus*. The predicted placental bacterial sources identified the oral cavity as a major contributor in one horse in this study, while the remaining samples’ sources were of unknown origin.

In humans, the taxonomic profile of the placental microbiome was discovered to be most similar to the oral microbiome, followed closely by the vaginal microbiome and further from the fecal microbiome [3]. This finding was found to be consistent with our study with pregnant mares earlier in gestation. *Gemella* was found to be increased in the oral cavity, and some were found in the placenta, while not in the feces and vagina, highlighting a potential similarity between these two body sites. *Gemella* has been previously found in the healthy equine mouth [35]. *Rikenellaceae* was found in all body sites, with the highest abundance within the feces, which is consistent with other equine microbiome studies [36,37]. *Campylobacter* was found in high levels within the vagina but not in the other body sites. *Campylobacter* has also been previously found in the healthy equine vaginal microbiome [18,21]. *Erysipelotrichaceae_UCG* was found in the feces and in small amounts in the placenta and vaginal samples. *Erysipelotrichaceae_UCG* has previously been isolated in bovine and zebra intestinal tract [38,39,40]. A study by Xia et al. 2017 examined full-term equine placentas and found similar results. Chorioallantois samples were similarly dominated by Firmicutes, Proteobacteria, Bacteroidetes, and Actinobacteria [23]. *Moraxella, Gemella*, and *Actinobacillus* were found in the oral cavity of the pregnant mare, which is congruent with our study in early gestation [23]. Additionally, *Campylobacter* in the vagina and *Ruminococcus* in the feces were other similarities between these equine studies [23]. Finally, late-term and the early gestation mares within this study shared *Streptococcus* among the placental samples [23], which had less ASV richness than the other body sites. The placental microbiome samples were all unique and host specific when characterized by richness and beta diversity, meaning the samples did not form a cohesive community. Beta dispersion demonstrates that the placenta samples are dissimilar to other placenta samples and have more host variation than other body sites. The feces and oral samples formed similar microbial communities with less variation than the placenta and vaginal samples. The increased beta dispersion suggests that the placenta harbors a diverse microbial community that is host specific. The vaginal samples also had large beta dispersion, which was expected because the vagina has been shown to have changes in composition associated with health, disease, and infections.

There were several limitations within this present study. It was found that endometrial samples taken by three different sampling strategies (swab, low volume lavage, and biopsy) all yielded similar microbiome results [41]. This highlights the potential for using swabs from uterine and extra-placental sites as a method to screen the maternal microbiome for health and disease in equine pregnancy. However, we recognize that whole placental tissues samples are often used for microbiome studies. In our investigations, we discovered this produces excess background noise from eukaryotic host contamination. Additionally, there has been much concern in the microbiome field about whether a placental microbiome exists. Recent studies challenge the placental microbiome dogma, specifically in humans. Using both PCR and Illumina sequencing, Lauder et al. matched a set of contamination controls to compare the healthy placental samples and found that the placental samples contained low and indistinguishable 16S rRNA copy numbers when compared with extraction blanks. In addition, using PERMANOVA of Bray–Curtis and UniFrac distances, no community separation was found [4]. Perez-Munoz et al. stated that their data do not support the existence of the microbiomes within the healthy fetoplacental environment. They further indicated that the current methodology (next generation sequencing) is faulty, the low biomass creates a sample too small to detect bacterial DNA accurately, and the potential for contamination is high and seen in numerous studies [42]. A follow up study completed by Leiby et al. examining placentas from spontaneous preterm births found no distinction between the background negative control and placenta samples [5]. A possible explanation from the previous authors is that the placenta is sterile until the rupture of the membranes during delivery. The microbiome found within cesarean sections is thought to be due to contamination, commonly from the DNA extraction kit [4]. There is a need to examine further every step of the sequencing process to assess the accuracy of the core microbiome of the placenta. Since the mares in this study were humanely euthanized, there was no contamination from rupture of membranes or birth. The uterus was aseptically removed in situ from the mare and opened. The swabs were sterilely collected and immediately frozen, and strict sterility and stringent bioinformatic filtering of the sequences was performed, including decontam package in R, as well as the SourceTracker package. A common issue in the analysis of microbiome communities is that they typically comprise several source “environments”, including different contaminants from sampling or DNA extraction, as well as other microbial communities that they interact with. The estimated bacterial sources from the placental samples, identified by the SourceTracker package in R, found the oral cavity to be the major source contributor in horse 1 and secondary in horses 2, 4, and 5. This is similar to a human study, which characterized the placenta being most closely related to the oral cavity [43]. The estimated primary source from horses 2, 3, 4, and 5 was of unknown bacterial origin. The environmental blanks were included in this analysis to give us confidence in the results. The percent contribution of the blanks in the placental samples ranged from 1% to, at most, 5% indicating that the contamination potential in these samples was low. The unknown source origin ranged from 6% to 88%, with the majority of the horses ranging from 62% to 78%. This suggests additional research is needed to further our knowledge of how these bacterial communities translocate to the placenta and interact with each other.

In our study using healthy pregnant mares, *Streptomyces* ssp. was isolated in one placental and one vaginal sample. *Pseudonocardia*, a close relative of *Amycolatopsis* and *Crossiella*, was isolated in two placental samples. The Pseudonocardiaceae family has been isolated in reproductive tracts of humans and cows [44,45]. A common pathology in pregnant mares involving the some of the bacteria identified in our study is placentitis. It is the leading cause of infectious abortion in the horse and contributes to roughly 19% of all abortions in the United States [46]. It causes episodic abortions in addition to weak and/or growth-restricted offspring [47]. There are four morphologic types of placentitis (ascending, nocardioform, diffuse, and multifocal) [34]. Ascending placentitis is the most common and is caused by bacteria ascending from the lower reproductive tract, and vaginal and fecal microbe contamination [34]. Organisms colonize the chorioallantois at the cervical star region, which results in the host producing pro-inflammatory cytokines and releasing prostaglandins that contribute to the cascade of events, leading to premature delivery of a foal [48]. The microorganisms associated with ascending placentitis include *Streptococcus equi subspecies zooepidemicus*, *Escherichia coli*, *Streptococcus equisimilis*, *Klebsiella pneumoniae*, and *Pseudomonas aeruginosa* [34]. Although the most common form of placentitis is ascending bacterial placentitis, other forms including diffuse and multifocal are caused by systemic infections [47] such as *Leptospira* sp. [34]. Focal or nocardioform placentitis involves colonization of the chorionic side of the chorioallantois by *Crossiella equi*, *Streptomyces* sp, and *Amycolatopsis species* (*Amycolatopsis kentuckyensis*, *Amycolatopsis lexingtonensis*, or *Amycolatopsis pretoriensis*) [46]. Nocardioform placentitis has an undefined etiology and may result from a uterine and/or placental microbial dysbiosis before or in early pregnancy, respectively. It would have been interesting to know the uterine microbiome of our mares prior to pregnancy because nocardioform pathogens were detected in the placenta. One could speculate that, if these mares were allowed to go to term, they may have developed nocardioform placentitis if an overgrowth progressed with gestation. Further longitudinal studies are warranted. 

Diagnosing placental infection during equine pregnancy is difficult since outward clinical signs are often not present. The ability to predict positively mares that will develop any type of placentitis would be valuable in equine practice. Understanding the relationship between the equine placenta and resident bacterial populations during healthy and diseased pregnancies could provide the opportunity to use extra-placental sources (oral, fecal, and/or vaginal) as biomarkers for predicting placentitis and associated adverse outcomes. It has been shown that there exists differences in the uterine microbiome between regions within the United States and between continents [49]. Therefore, we chose to characterize only pregnant mares from the same location to reduce variables such as a climate, forage, and feed, etc. that may influence the pregnant microbiome. Another limitation may be the specific placental area that was sampled. In studies of experimental placentitis, sampling allantoic fluid from pregnant mares has been used antemortem to assess the degree of fetoplacental infection and response to treatment [50]. While sampling allantoic fluid is an invasive procedure that is not routinely performed clinically, our study swabbed the allantoic surface to avoid contamination that might happen during a transabdominal allantocentesis. Furthermore, Fedorka et al. noted structural changes in the allantoic portion of placentas with nocardioform placentitis [46]. In that study, a large number of placentas diagnosed with nocardioform placentitis had notable allantoic hyperplasia, with a subset of these being cystic, and is believed to occur because of chronic inflammation [46]. 

Further investigations are required to evaluate clinical cases of equine placentitis as well as the mare’s uterine microbiome before pregnancy. Rapid screening of microbial biomarkers from extra-placental sites that can be sampled non-invasively may improve our ability to detect pregnant mares that will develop pathology before clinical signs are present. Identification of bacterial targets in extra-placental body sites as causative in adverse pregnancy outcomes would revolutionize the way we manage pregnancy in the mare. 

## 5. Conclusions

In conclusion, metagenetics did reveal distinct community differences in the oral, fecal, vaginal, and placental cavities of the horse. We believe there is rationale to challenge the dogma of the placenta being sterile in horses. The equine placenta did show similarities in its microbial communities to the oral cavity when assessing alpha diversity. At the genus level, some similarities were noted between body sites including *Gemella* and *Porphyromonas*. The placenta harbored its own unique microbiome in relation to diversity, relative abundance, and bacterial richness. The placenta was dominated by *Gemella*, *Rikenellaceae_RC9*, *Porphyromonas*, and *Streptococcus*. The predicted placental bacterial sources identified the oral cavity as a major contributor in one horse in this study, while the remaining samples’ sources were of unknown origin. The high proportions of unknowns and low proportions of blanks helped us determine that the potential of contamination in these samples was low. Further research needs to be completed to investigate how bacteria are translocated to the placenta from these other body sites and how they contribute to the development of placentitis. Continuation of this study would involve development of a screening method using next generation sequencing to identify microbial community dysbiosis rapidly to monitor mares that could potentially develop placentitis.

## Figures and Tables

**Table 1:** Age and gestational day of mares.

ID	Age (Years)	Gestation (Days)
Horse 1	8	96–98
Horse 2	8	98–100
Horse 3	6	100–102
Horse 4	4	98–99
Horse 5	5	98–126

## Data Availability

The data presented in this study are openly available in the National Center for Biotechnology Information Sequence Read Archive, reference number BioProject ID PRJNA945405.
